# Inhibition of the Interaction Between Group I Metabotropic Glutamate Receptors and PDZ-Domain Proteins Prevents Hippocampal Long-Term Depression, but Not Long-Term Potentiation

**DOI:** 10.3389/fnsyn.2019.00013

**Published:** 2019-04-18

**Authors:** Sergey Neyman, Karl-Heinz Braunewell, Kara E. O’Connell, Kumlesh K. Dev, Denise Manahan-Vaughan

**Affiliations:** ^1^Department of Neurophysiology, Medical Faculty, Ruhr University Bochum, Bochum, Germany; ^2^Drug Development, School of Medicine, Faculty of Health Sciences, Trinity College Dublin, Dublin, Ireland

**Keywords:** DHPG, synaptic plasticity, mGlu receptor, mGlu1, mGlu5, CA1, tamalin

## Abstract

The group I metabotropic glutamate (mGlu) receptor subtypes, mGlu1 and mGlu5, strongly regulate hippocampal synaptic plasticity. Both harbor PSD-95/discs-large/ZO-1 (PDZ) motifs at their extreme carboxyl terminals, which allow interaction with the PDZ domain of Tamalin, regulate the cell surface expression of group I mGlu receptors, and may modulate their coupling to signaling proteins. We investigated the functional role of this interaction in hippocampal long-term depression (LTD). Acute intracerebral treatment of adult rats with a cell-permeable PDZ-blocking peptide (pep-mGluR-STL), designed to competitively inhibit the interaction between Tamalin and group 1 mGlu receptors, prevented expression of LTD in the hippocampal CA1 region without affecting long-term potentiation (LTP) or basal synaptic transmission. Pep-mGluR-STL prevented facilitation by the group I mGlu receptor agonist, (S)-3,5-Dihydroxyphenylglycine (DHPG), and the mGlu5 agonist, (R,S)-2-chloro-5-Hydroxyphenylglycine (CHPG), of short-term depression (STD) into LTD, suggesting that Tamalin preferentially acts by mediating signaling through mGlu5. These data support that Tamalin is essential for the persistent expression of LTD and that it subserves the effective signaling of group 1 mGlu receptors.

## Introduction

Metabotropic glutamate (mGlu) receptors are widely distributed throughout the CNS, and play a crucial role in glutamate-mediated neurotransmission and synaptic plasticity events. Based on molecular, signaling and pharmacological similarities, the mGlu receptor subtypes, mGlu1–mGlu8, have been divided into three groups (I–III). The persistence of hippocampal synaptic plasticity and memory is strongly regulated by group I mGlu receptors (mGlu1 and mGlu5; Cohen and Abraham, 1996; Manahan-Vaughan, 1997; Cohen et al., 1998; Balschun et al., 1999; Naie and Manahan-Vaughan, 2004, 2005; Mukherjee and Manahan-Vaughan, 2013). Group I mGlu receptors are positively coupled to phospholipase C and couple to inositol 1,4,5,-trisphosphate/Ca^2+^ signal transduction (Abe et al., 1992; Aramori and Nakanishi, 1992; Nakanishi et al., 1998). The pharmacological activation of these receptors triggers protein synthesis-dependent chemical depression in the CA1 region of young rats (Huber et al., 2001), facilitates short-term potentiation into long-term potentiation (LTP; Cohen and Abraham, 1996; Manahan-Vaughan and Reymann, 1996; Manahan-Vaughan et al., 1996; Cohen et al., 1998) and facilitates short-term depression (STD) into long-term depression (LTD; Popkirov and Manahan-Vaughan, 2011). By contrast, the blockade of group I mGlu receptors using pharmacological antagonists prevents hippocampus-based spatial memory, impairs late-LTP (Balschun et al., 1999; Naie and Manahan-Vaughan, 2004, 2005; Hagena and Manahan-Vaughan, 2015) and inhibits hippocampal LTD (Manahan-Vaughan, 1997; Popkirov and Manahan-Vaughan, 2011; Goh and Manahan-Vaughan, 2013a; Hagena and Manahan-Vaughan, 2015).

PSD-95/Discs large/ZO-1 (PDZ) domain-containing proteins are known to interact with PDZ-binding motifs located at the extreme three residues of the carboxyl terminus of target proteins (Garner et al., 2000; Dev et al., 2001). These interactions provide a molecular mechanism for clustering receptors at the plasma membrane, allowing for receptor cross-talk, and directing kinases and phosphatases toward their substrates. Group I mGlu receptors are localized on postsynaptic neuronal membranes as constitutive dimers (Craven and Bredt, 1998; Kunishima et al., 2000; Kniazeff et al., 2004; Hlavackova et al., 2005). The cell-surface expression and clustering of mGlu receptors, as well as their coupling to signaling proteins, is enabled by proteins containing PDZ domains (Craven and Bredt, 1998; Sheng and Sala, 1998; Garner et al., 2000; Dev et al., 2001). Tamalin is a PDZ domain-containing protein that interacts with group I mGlu receptors (Kitano et al., 2002; Kniazeff et al., 2004). It forms a complex with mGlu receptors and guanine nucleotide exchange factor cytohesins and promotes intracellular trafficking and cell surface expression of group 1 mGlu receptors (Kitano et al., 2003). This protein interacts with the intracellular carboxyl terminal located PDZ binding motifs with mGlu1 (-KQSSSTL) and mGlu5 (-TQSSSSL). Tamalin may play a key role in connecting group I mGlu receptors to the GTP-binding proteins that regulate intracellular protein transport and synaptic organization (Kitano et al., 2002).

We and others have previously shown that short peptides containing the PDZ motifs of glutamate receptors can competitively block their interactions with PDZ domain-containing proteins to regulate receptor function in hippocampal slice cultures and *in vivo* (Daw et al., 2000; Aarts et al., 2002; Hirbec et al., 2003). Here, we investigated whether the *in vivo* infusion of a cell-permeable PDZ peptide, that inhibits the interaction between Tamalin and group I mGlu receptors, alters synaptic plasticity in the hippocampus and whether this peptide influences synaptic plasticity that is strengthened by pharmacological activation of group I mGlu receptors.

## Materials and Methods

The present study was carried out in accordance with the European Communities Council Directive September 22nd, 2010 (2010/63/EU) for care of laboratory animals and after approval of the ethics committee of the local (state) government (Landesamt für Naturschutz, Umweltschutz und Verbraucherschutz, Nordrhein Westfalen). All efforts were made to minimize the number of animals used.

### Pull-Down Studies

The PDZ domain (Val^100^-Ser^190^) of Tamalin was subcloned into pMALc2X (New England Biolabs, Beverly, MA, USA), while the last seven residues of the C-terminal domains of mGlu1 (-KQSSSTL) and mGlu (-TQSSSSL) were subcloned into pGEX-4T-1 (Pharmacia, Uppsala, Sweden). Glutathione-S-transferase (GST) pull-down experiments were performed as described previously (Dev et al., 1999, 2000). Briefly, maltose binding protein (MAL) and GST-fused proteins were purified from transformed *Escherichia coli* strain, BL21, and lysates were prepared by sonication and solubilization in ice-cold PTxE buffer [phosphate-buffered saline (PBS), 1% Triton X-100, 0.1 mM EDTA, pH 7.4]. The lysates containing GST-mGlu1 (-KQSSSTL), or mGlu5 (-TQSSSSL), were rotated with 20 μl glutathione Sepharose 4B beads (Pharmacia, Uppsala, Sweden) in the presence of 1 mg/mL bovine serum albumin (BSA, Sigma) for 30 min at 4°C, after which the coupled Sepharose beads were washed with 2 × 1 ml aliquots of PTx buffer (PBS, 0.1% Triton X-100, pH 7.4). The beads were then resuspended in PTx buffer and incubated with lysates containing MAL-PDZ Tamalin in the presence of 1 mg/mL BSA, with or without the synthetic peptides: the last seven residues of the carboxy terminal region of mGlu1 (-KQSSSTL), mGlu5 (-TQSSSSL), or a PDZ motif deleted control (-KQSS). After rotation for 4 h at 4°C, the bead suspensions were washed with 3 × 1 ml aliquots of PTx buffer, resuspended in 30 μl PTx buffer. Dot blot arrays were performed using a monoclonal anti-MAL antibody (M6295, MBP-17, Sigma, St. Louis, MO, USA) to determine the level of MAL-PDZ Tamalin retained, as described previously (O’Connell et al., 2011; Shrestha et al., 2014). The dot immunoblots were performed in two separate array sets (in duplicates), so that the data could be subjected to 2-dimensional (2D) normalization i.e., normalization to remove both dot-to-dot and blot-to-blot variation, as described previously (Chatterjee et al., 2009; O’Connell et al., 2011; Shrestha et al., 2014).

### Generation of Cell-Permeable pep-mGluR-STL

A synthetic cell-permeable peptide (pep-mGluR-STL) was created based on the sequence of the last seven amino acids of mGlu1 (-KQSSSTL, *the PDZ binding motif underlined*) which also closely matches mGlu5 (-TQSSSSL) to inhibit the interaction between group I mGlu receptors and Tamalin. Specifically, the last seven residues of the carboxyl terminal region of mGlu1 (-KQSSSTL) was fused to the cell-membrane transduction domain of the human immunodeficiency virus-type 1 (HIV-1) Tat protein (YGRKKRRQRRR) to render the 18 amino acid peptide cell-permeable, pep-mGluR-STL (YGRKKRRQRRR-KQSSSTL), based on previous reports (Healy et al., 2013). A peptide with the last three residues deleted (i.e., without the PDZ binding motif) was used as control, pep-mGlu-con (sequence YGRKKRRQRRR-KQSS). Non-labeled peptides were synthesized to 85% for greater purity. Peptides were resuspended in saline at a final concentration of 5 mg/ml and stored at −80°C in aliquots until use. Peptides were custom-purchased from Sigma-Aldrich (Arklow, Co. Wicklow, Ireland).

### *In vivo* Treatment With pep-mGluR-STL Prior to *in vitro* Analysis

Under anesthesia, using previously described methods (Manahan-Vaughan, 1997), a cannula was implanted into the lateral cerebral ventricle of 7–8-week-old male Wistar rats. After 8–10 days of recovery from surgery, pep-mGluR-STL was administered to inhibit interactions between Tamalin and group I mGlu receptors. One hour prior to hippocampal dissection, animals were injected with 5 μl (25 μg) or 10 μl (50 μg) of pep-mGluR-STL (5 mg/ml) over a 5 min period. In control experiments, animals were treated with a control peptide lacking the PDZ binding motif [the last three residues (–STL)]. Injection was enabled by means of a guide cannula attached to flexible polyethylene tubing, which was inserted into a Hamilton syringe. The animals could move freely during the injection procedure.

### *In vitro* Electrophysiology

One hour after peptide injection, the rats were anesthetized with ether and then decapitated. Brains were dissected in ice-cold Ringer’s solution (as described below). Immediately after preparation, the slices (400 μM) were placed on a nylon net in a 2 ml-circulation chamber at the interface between incubation medium and a humidified atmosphere of 95% O_2_/5% CO_2_ and continuously perfused (with a constant flow rate of 3 ml/min) with an oxygenated Ringer’s solution (in mM: NaCl 124, KCl 4.9, KH_2_PO_4_ 1.2, MgSO_4_ 1.3, CaCl_2_ 2.5, NaHCO_3_ 25.6, D-Glucose 10) at 35°C. Following 1.5 h equilibration, the slices were submerged by filling the chamber to a volume of 3 ml with warmed (35°C) O_2_/CO_2_ Ringer’s solution. The flow rate was then adjusted to 1–1.5 ml/min. Monopolar platinum-tipped silver chloride electrodes were positioned in the Stratum radiatum of the CA1 region for stimulation, and in the CA1 dendritic area for recording. Typically recordings were obtained from two slices simultaneously: one hippocampus was used in the experiments described below, the other slice received test-pulse stimulation to verify slice viability (data not shown). In cases where unstable responses were obtained from the latter slice, data from the former slice were excluded from analysis. In the “Results” section the cohort numbers (n’s) provided correspond to one slice per animal that was included in the analysis.

Prior to starting the experiment, a stimulus-response (input/output) relationship was obtained by stimulating the hippocampal slice in the range of 60 through 600 μA. The slope of the field excitatory postsynaptic potential (fEPSP) was measured as the maximum slope through the five steepest points obtained on the first positive deflection of the potential. A stimulation intensity that evoked 50% of the maximal fEPSP obtained was used for all subsequent experiments. Each time point was determined by averaging five responses that were evoked at 0.025 Hz (0.2 ms stimulus duration; 16,000 Hz sample rate).

Basal synaptic transmission was recorded for 30 min and, without changing the stimulation intensity, patterned stimulation was applied to induce synaptic plasticity (see below). The time-points of the initial 30 min of each experiment were obtained at 5 min intervals. This interval was continued until 15 min after an attempt to induce synaptic plasticity (i.e., 45 min after starting recordings), thereupon the time-point interval was changed to 15 min. In cases where no patterned stimulation was given, test-pulses were applied for 4.5 h in total at the same time-point intervals as were used for plasticity experiments.

LTP was induced with high frequency stimulation (HFS) comprising three stimulus trains of 100 pulses at 5-min intervals. STD was induced with low frequency stimulation (LFS) at 1 Hz (900 pulses), whereas persistent LTD was induced with LFS at 2 Hz (1,200 pulses). In pharmacological experiments, the mGlu5 receptor agonist, (S)-3,5-Dihydroxyphenylglycine (DHPG; Biozol, Eching, Germany), the mGlu5 receptor agonist, (R,S)-2-chloro-5-Hydroxyphenylglycine (CHPG; Biozol, Eching, Germany), or the mGlu5 receptor antagonist 2-methyl-6-(phenylethynyl)pyridine (MPEP; Biozol, Eching, Germany), were applied for 20 min prior to LFS.

To assess for statistical significance, an analysis of variance (ANOVA) with repeated measures (ANOVA) was applied to test for between-group or within-group effects. The time-points included corresponded to all time points after application of HFS or LFS. In the case of the baseline experiment (shown in Figure 3A) all data points were included in the analysis. A Student’s *t*-test was applied *post hoc* in some cases to determine the time-point of onset of a change in synaptic plasticity. All data are reported as the mean ± standard error of the mean. The level of significance was set at *p* < 0.05.

**Figure 1:**
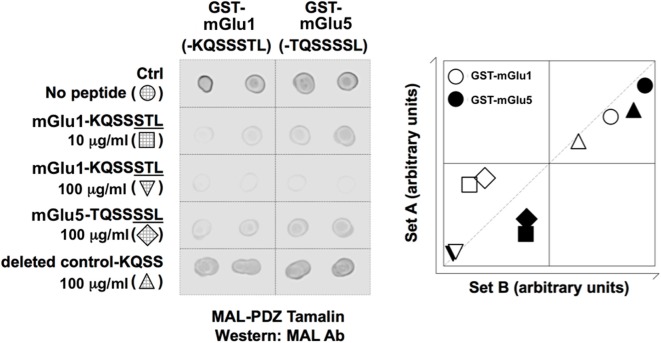
Blocking peptides (pep-mGluR1 and 5) inhibit interaction between mGlu1- or mGlu5 receptors and Tamalin. *Left*: dot blot shows that mGlu1-KQSSSTL and mGlu5-TQSSSSL peptides inhibit interaction between GST-mGlu1 (-KQSSSTL) and GST-mGlu5 (-TQSSSSL). Abbreviations: Ab, antibody; Ctrl, control; GST, glutathione-S-transferase; MAL, maltose binding protein; PDZ, PSD-95/discs-large/ZO-1. *Right*: a scatter plot for each spot was obtained by plotting the density in the first array experiment (*x*-axis) against that obtained in the second array experiment (*y*-axis). The samples lying along the *x/y* axis indicated consistency between array experiments. The samples lying at the extremities in this analysis displayed the highest interactions.

**Figure 2:**
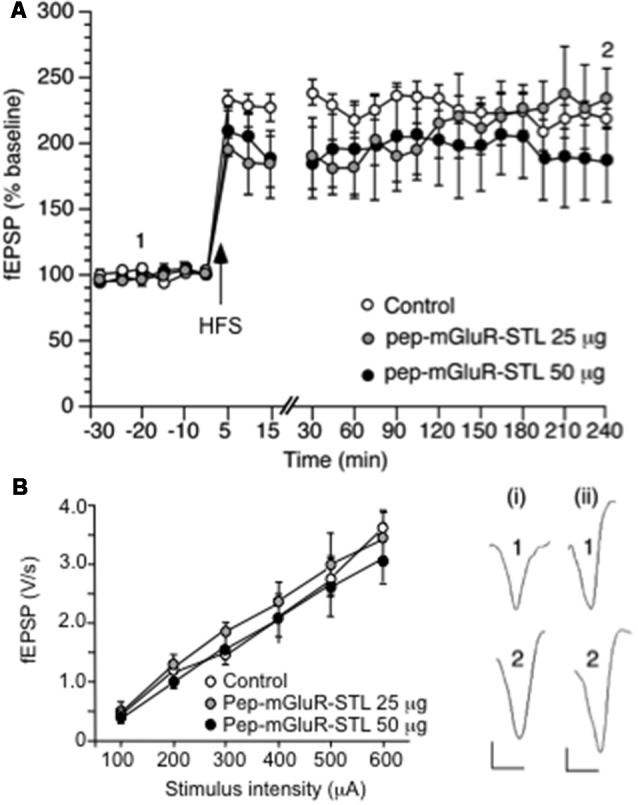
Long-term potentiation (LTP) and the stimulus-response relationship are not altered by pep-mGluR-STL. **(A)** High frequency stimulation (HFS) induces robust LTP that lasts for at least 4 h in the CA1 region of hippocampal slices from animals that were treated with control peptide (*n* = 8). Treatment of animals with pep-mGluR-STL (25 μg, *n* = 5, or 50 μg, *n* = 5), has no effect on the expression of LTP. Line-break indicates change in time-scale. Analogs represent responses elicited in the presence of: (i) control peptide; and (ii) pep-mGluR-STL (25 μg) at the time-points noted. Vertical bar: 1 mV, Horizontal bar: 10 ms. **(B)** Comparison of the stimulus–response (input/output) relationship in hippocampi from animals that had been treated with control peptide, 25 μg or 50 μg of pep-mGluR-STL (all *n* = 7) revealed no differences in field excitatory postsynaptic potential (fEPSP) responses that were evoked in the range of 60–600 μA.

**Figure 3:**
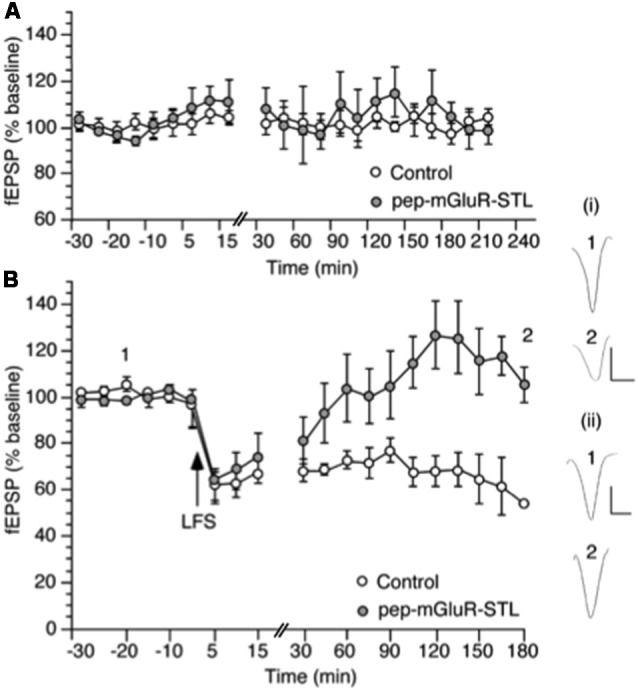
Pep-mGluR-STL prevents persistent long-term depression (LTD) in the hippocampal CA1 region. **(A)** Basal synaptic transmission is not affected by the prior application of pep-mGluR-STL (25 μg, *n* = 5) compared to responses evoked in hippocampal slices from animals that had been treated with control peptide (*n* = 5). **(B)** Low frequency stimulation (LFS, 2 Hz, 1,200 pulses) induces persistent LTD which lasts for at least 3 h in the CA1 region of hippocampal slices from animals that were treated with control peptide (*n* = 6). Treatment with pep-mGluR-STL (25 μg, *n* = 6) significantly prevents the expression of LTD. Line-breaks indicate changes in time-scale. Analogs represent responses elicited in the presence of: (i) control peptide; and (ii) pep-mGluR-STL at the time-points noted. Vertical bar: 1 mV, Horizontal bar: 10 ms.

## Results

### Tamalin Interacts With mGlu1 and mGlu5 Receptors

To confirm interactions between mGlu1, or mGlu5 receptors, and Tamalin, GST pull-down experiments were conducted in the presence and absence of synthetic peptides: the last seven residues of the carboxy terminal region of mGlu1 (-KQSSSTL), mGlu5 (-TQSSSSL), or a PDZ motif deleted control (-KQSS). We observed an interaction between GST-mGlu1-KQSSSTL and MAL-PDZ Tamalin as well as GST-mGlu5-TQSSSSL and MAL-PDZ Tamalin (Figure 1), in agreement with previous reports (Kunishima et al., 2000; Kitano et al., 2002, 2003). Using similar blocking peptide study approaches, as described previously (Daw et al., 2000; Hirbec et al., 2003), we demonstrated that these interactions were inhibited by the peptides mGlu1-KQSSSTL and mGlu5-TQSSSSL, but not by the PDZ motif deleted control (-KQSS; Figure 1). The data demonstrate that the last seven residues of the carboxy terminal region of mGlu1 (-KQSSSTL) can be utilized to inhibit the interaction between Tamalin and mGlu receptors, as was conducted in the present study.

### pep-mGluR-STL Does Not Alter Long-Term Potentiation in Hippocampal CA1 Synapses

HFS (three stimulus trains of 100 Hz at 5 min intervals) evoked robust LTP that was still present at the end of the 4 h recording period (*n* = 8, Figure 2A). Application of 25 μg (*n* = 5) or 50 μg (*n* = 5) of pep-mGluR-STL had no effect on the expression of LTP (ANOVA, 25 μg: within factor *F*_(1,17)_ = 0.659, *p* = 0.8397; between factor *F*_(1,17)_ = 1.278, *p* = 0.2846; ANOVA, 50 μg: within factor *F*_(1,17)_ = 0.807, *p* = 0.6831; between factor*F*_(1,17)_ = 0.244, *p* = 0.9992).

Comparison of the stimulus-response (input/output) relationship in naive slices revealed no significant differences in fEPSP responses evoked in the range of 100 through 600 μA (*n* = 7 each, Figure 2B, Three-way ANOVA: between-factor *F*_(2,17)_ = 1.61, *p* = 0.7114). Basal synaptic transmission was also unaffected by the peptide (Figure 3A, ANOVA: within factor *F*_(1,17)_ = 0.711, *p* = 0.7429; between factor *F*_(1,17)_ = 0.327, *p* = 0.538; all *n* = 5).

### Persistent Long-Term Depression in Hippocampal CA1 Is Inhibited by pep-mGluR-STL

LFS at 2 Hz (1,200 pulses) induced persistent LTD that was still present at the end of the 4 h recording period in hippocampi from animals that had been treated with control peptide (*n* = 6, Figure 3B). Treatment with 25 μg of pep-mGluR-STL (*n* = 6) significantly inhibited the expression of LTD compared to LTD in controls (ANOVA: within factor *F*_(1,19)_ = 2.581, *p* = 0.003; between factor *F*_(1,19)_ = 1.978, *p* = 0.025). Effects became apparent 105 min after LFS had been applied (*t*-test, *p* < 0.05). The response profile was similar to the impairment of LTD by mGlu5 receptor antagonism (Neyman and Manahan-Vaughan, 2008).

### Facilitation of LTD Induced by Pharmacological Activation of mGlu5 Is Prevented by pep-mGluR-STL

**Figure 4:**
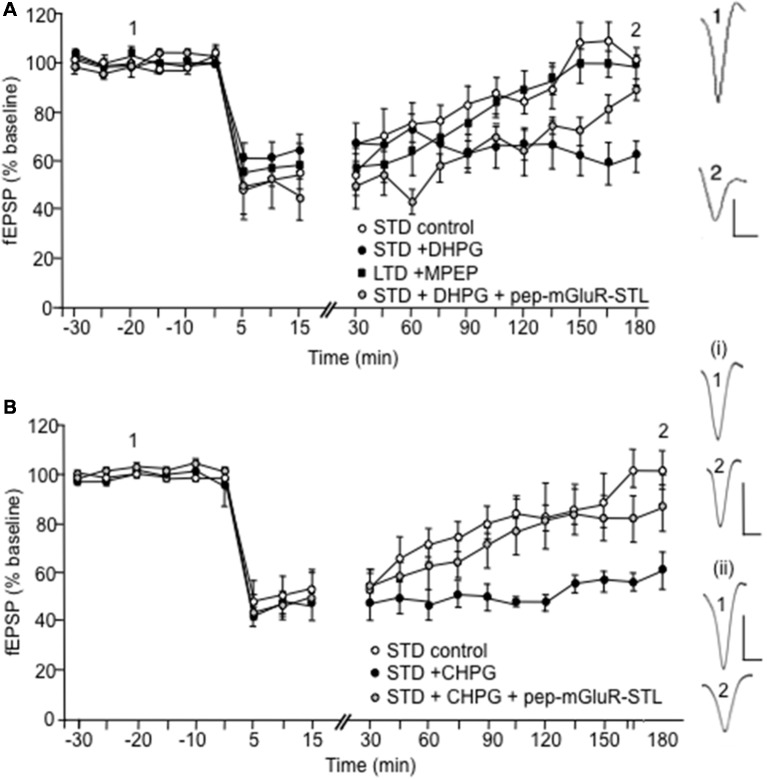
Facilitation of LTD induced by pharmacological activation of group I mGlu receptors is prevented by pep-mGluR-STL. **(A)** LFS in a frequency of 1 Hz (900 pulses) induces short-term depression (STD) that lasts for up to 3 h in the CA1 region of hippocampal slices from animals that were treated with control peptide (*n* = 6). Application of (S)-3,5-Dihydroxyphenylglycine (DHPG; 5 μM) prior to LFS (*n* = 6) facilitates STD into LTD. The facilitating effect of DHPG on LTD is prevented by prior treatment of animals with pep-mGluR-STL (25 μg, *n* = 6). Induction of LTD that lasts for longer than 3 h by stronger LFS (2 Hz, 1,200 pulses) is prevented in hippocampal slices from by the mGlu5 receptor antagonist, MPEP (40 μM, *n* = 6). Analogs to the right of the graph represent responses elicited in the presence of DHPG in blocking peptide-treated hippocampi at the time-points noted. Vertical bar: 1 mV, Horizontal bar: 10 ms. **(B)** Application of (R,S)-2-chloro-5-Hydroxyphenylglycine (CHPG; 100 μM) prior to LFS (1 Hz, 900 pulses, *n* = 8) facilitates STD into LTD, compared to controls (*n* = 7) The facilitating effect of CHPG on LTD of hippocampal slices from is prevented by prior treatment of animals with pep-mGluR-STL (25 μg, *n* = 8). Line-breaks indicate changes in time-scale. Analogs to the right of the graph represent responses elicited in the presence of: (i) CHPG in blocking peptide-treated hippocampi; and (ii) CHPG in control peptide-treated hippocampi at the time-points noted. Vertical bar: 1 mV, Horizontal bar: 10 ms.

To clarify whether the pep-mGluR-STL effects were mediated by a change in group I mGlu receptor contributions to LTD, we then examined its effects on the late component of LTD that is supported by activation of group I receptors (Figure 4).

LFS at a frequency of 1 Hz (900 pulses) produced STD (*n* = 6, Figure 4A). Application of the group 1 mGlu receptor agonist (S)-DHPG (5 μM) facilitated STD into LTD that lasted for at least 4 h compared to control STD (Figure 4A, *n* = 6; ANOVA: within factor *F*_(1,13)_ = 5.458, *p* = 0.0001; between factor *F*_(1,13)_ = 4.512, *p* = 0.0001). LFS (1 Hz) in the presence of DHPG, when applied to the hippocampi of animals that had been treated with pep-mGluR-STL (25 μg, *n* = 6) resulted in a significant impairment of LTD (ANOVA: within factor *F*_(1,13)_ = 4.938, *p* = 0.0001; between factor *F*_(1,13)_ = 2.979, *p* = 0.001, compared to the LFS/DHPG condition; Figure 4A). A three-way ANOVA with repeated measures confirmed the significance of effects (within factor *F*_(2,27)_ = 3.595, *p* = 0.0001; between factor *F*_(2,27)_ = 8.703, *p* = 0.001).

DHPG preferentially activates mGlu5 receptors. To examine the profile of LTD that is induced by stronger LFS (2 Hz, 1,200 pulses) and confirm that mGlu5 contributes to the stabilization of the later phase of LTD under the current experimental conditions, we treated the slices with the selective mGlu5 receptor antagonist, MPEP (40 μM, *n* = 6). Here the late phase of LTD was prevented, as was shown previously (Neyman and Manahan-Vaughan, 2008). The profile of the response was significantly different from the response obtained when LFS (1 Hz) was applied in the presence of pep-mGluR-STL and DHPG, however, suggesting that both mGlu1 and mGlu5 receptor contributions to LTD may be affected by the blocking peptide (ANOVA: within factor *F*_(1,13)_ = 5.239, *p* = 0.0001; between factor *F*_(1,13)_ = 3.467, *p* = 0.0001).

To clarify whether the mGlu5 receptor was the primary target of the pep-mGluR-STL-mediated impairment of LTD shown in Figure 4, we then applied the selective mGlu5 receptor antagonist CHPG (100 μM, Figure 4B) and tested its effect on STD induced by LFS (1 Hz, 900 pulses) in the presence or absence of pep-mGluR-STL. CHPG application (*n* = 8) resulted in facilitation of LTD compared to controls (*n* = 7; within factor *F*_(1,19)_ = 2.698, *p* = 0.001; between factor *F*_(1,19)_ = 13.361, *p* = 0.001). The facilitation of STD into LTD by CHPG was prevented by pep-mGluR-STL (*n* = 8, Figure 4B; ANOVA: between factor *F*_(1,13)_ = 11.317, *p* = 0.0001). This suggests that pep-mGluR-STL may mediate its effects by predominantly affecting mGlu5 receptor function.

## Discussion

PDZ motif-containing peptides have been used as powerful tools to competitively block specific PDZ interactions and to understand their functional roles (Daw et al., 2000; Dev et al., 2000; Aarts et al., 2002). Based on these strategies, and since both mGlu1 and mGlu5 receptors harbor similar PDZ binding motifs, we generated a synthetic peptide containing PDZ binding motif of mGlu1 (-KQSSSTL, *the PDZ binding motif underlined*), which also closely matches mGlu5 (-TQSSSSL), to competitively inhibit the interaction between Tamalin and group I mGlu receptors (pep-mGlu-STL). Our data shows that cerebral infusion of pep-mGlu-STL inhibited LTD in the hippocampal CA1 region. Moreover, a control peptide lacking the PDZ binding motif, namely the last three residues (–STL), showed no effects on synaptic plasticity indicating the requirement of a PDZ binding motif. We also observed a lack of effect of pep-mGluR-STL on LTP, which could reflect a non-critical role for Tamalin in plasticity processes that involve strengthening of synaptic weights. Alternatively, the lack of effect of pep-mGluR-STL on LTP could be due to a frequency dependency of mGlu-participation in LTP (Wilsch et al., 1998), such that mGlu activation becomes critical for LTP persistency only under conditions of intense afferent stimulation. Notably, the regulation of LTD (but not LTP) by pep-mGluR-STL indicates the potential use-dependent effects of PDZ blocking peptides.

Tamalin is a scaffold protein that comprises multiple protein-interacting domains (Nevrivy et al., 2000; Kitano et al., 2003). Co-immunoprecipitation using brain extracts revealed an interaction between Tamalin and group I (but not group II) mGlu receptors (Kitano et al., 2002). Tamalin contains leucine-zipper regions that bind to the carboxy-terminal tails of mGlu receptors, and it also contains a coiled-coil region that interacts with the guanine nucleotide exchange factor cytohesins (Nevrivy et al., 2000; Kitano et al., 2002). Moreover, Tamalin associates with a range of scaffold proteins (Kitano et al., 2002, 2003). Tamalin can function as a signaling molecule that mediates spleen tyrosine kinase (Syk) signaling in COS-7 cells, and additionally promotes phosphorylation at tyrosine residues of the immunoreceptor tyrosine-based activation motif (ITAM) sequences by the Src family of kinases (Hirose et al., 2004). Taken together, these data provide a molecular link for Tamalin in regulating receptor phosphorylation and trafficking and also in altering postsynaptic organization within neurons.

Activation of mGlu1 receptors results in an increase in intracellular Ca^2+^ concentration, depolarization of CA1 pyramidal neurons and an increased frequency of spontaneous inhibitory postsynaptic potentials (Mannaioni et al., 2001). Furthermore, mGlu1 receptor activation in LTD results in a reduction in the surface expression of the α-amino-3-hydroxy-5-methyl-4-isoxazole propionic acid (AMPA) receptor GluA1 subunit (Xiao et al., 2001). Activation of mGlu5 receptors results in suppression of the Ca^2+^-activated potassium current and a potentiation of N-methyl-D-aspartate (NMDA) receptor currents (Jia et al., 1998; Attucci et al., 2001; Mannaioni et al., 2001; Volk et al., 2006). Differences have been described in the regulation by mGlu1 and mGlu5 receptors of LTD (Jia et al., 1998; Naie et al., 2007), whereby activation of mGlu1 receptors during (but not after) LFS is a critical step for LTD to endure for more than 2–3 h (Neyman and Manahan-Vaughan, 2008). By contrast, activation of mGlu5 receptors after (but not during) LFS is required for lasting LTD (Neyman and Manahan-Vaughan, 2008). In the dentate gyrus, LTD induced by LFS is prevented by antagonism of mGlu1, but not mGlu5 receptors, whereas both receptors contribute to chemical LTD (Huber et al., 2001; Naie et al., 2007). In the CA1 region, mGlu1 receptors mediate expression of chemical LTD (Volk et al., 2006), whereas very persistent ( >24 h) LTD that is elicited by LFS in the CA1 region *in vivo* is mGlu5-receptor-dependent, as is LTD ( >24 h) that is facilitated by spatial learning (Popkirov and Manahan-Vaughan, 2011; Goh and Manahan-Vaughan, 2013b). In addition, LTD in the CA1 region requires protein synthesis (Manahan-Vaughan et al., 2000), whereas in the dentate gyrus it does not (Pöschel and Manahan-Vaughan, 2007). These findings suggest that a number of mechanistically distinct forms of LTD are expressed in the hippocampus, and what is particularly striking, in this regard, is that all forms of LTD that have been identified to date are regulated in a differentiated way by group I mGlu receptors (Huber et al., 2001; Volk et al., 2006; Naie et al., 2007; Neyman and Manahan-Vaughan, 2008; Mukherjee and Manahan-Vaughan, 2013; Hagena and Manahan-Vaughan, 2015). Interestingly, and in line with this ostensible differentiation in control by group I mGlu receptors of distinct forms of hippocampal LTD, we found that pep-mGluR-STL inhibited LFS-mediated LTD, as well as the facilitation of STD into LTD elicited by the group I mGlu receptor agonist, DHPG (Ito et al., 1992; Schoepp et al., 1994) and the mGlu5 receptor agonist, CHPG (Doherty et al., 1997). Since both agonists preferentially activate mGlu5 in the CA1 region (Palmer et al., 1997; Huber et al., 2001; Volk et al., 2006), these results suggest that under the experimental conditions tested here, the interaction between Tamalin and mGlu5, rather than mGlu1 receptors, may play an important role in the development of LTD. It also suggests that the differentiated contribution of group I mGlu receptors to hippocampal LTD may comprise a key determinant of the magnitude, persistence and purpose of the LTD induced. This suggestion is supported by studies of the group I mGlu receptor-dependency of LTD that is promoted by spatial learning in rodents (Popkirov and Manahan-Vaughan, 2011; Goh and Manahan-Vaughan, 2013b; Dietz and Manahan-Vaughan, 2017). This property is particularly interesting in light of the reported role of different kinds of hippocampal LTD in the encoding of distinct forms of spatial learning (Manahan-Vaughan and Braunewell, 1999; Kemp and Manahan-Vaughan, 2004, 2008, 2012; Goh and Manahan-Vaughan, 2013a).

It is notable that although the blocking peptide was designed to contain the PDZ binding motif of the mGlu1 receptor, the effects we observed on LTD were mediated primarily by mGlu5 receptors. The PDZ binding motif of mGlu1 (-KQSSSTL) is similar to the PDZ binding motif of the mGlu5 receptor (-TQSSSSL), which may explain why mGlu5 receptor functions were specifically affected. Nonetheless, we cannot exclude that the sequence peptides may bind to a number of PDZ-domain-containing proteins. Here, Na^+^/H^+^ exchanger regulatory factor-2 (NHERF-2) is a possible candidate (Paquet et al., 2006). While taking this into account, it seems likely that the predominant mode of action of pep-mGluR-STL involves disruption of key contributions of mGlu5 to hippocampal LTD: the peptide prevented the facilitation of STD into LTD that is enabled by selective activation of mGlu5 receptors with CHPG. The profile of the inhibition was albeit different from the profile of LTD inhibition by the mGlu5 receptor antagonist, MPEP, suggesting that an involvement of mGlu1 receptor cannot be entirely ruled out.

The molecular mechanisms underlying the inhibition of LTD by pep-mGluR-STL are not likely to comprise an interference with the regulation of NMDA receptor function by mGlu5 receptors (Jia et al., 1998; Attucci et al., 2001; Mannaioni et al., 2001; Volk et al., 2006), or mGlu1 receptors (Benquet et al., 2002; Heidinger et al., 2002) by virtue of the fact that the deficits in LTD emerged after the first 15 min of synaptic depression. Antagonism of NMDA receptors results in an impairment of CA1 LTD that becomes apparent immediately after the conclusion of LFS (Manahan-Vaughan, 1997). This is consistent with the well-documented membrane voltage-dependent entry of Ca^2+^ through NMDA receptors that is a key step for the induction of LTD in the CA1 region (Mulkey and Malenka, 1992). Given that our data support that mGlu5 receptors were most particularly affected by pep-STL-mGluR, it is possible that mGlu1 receptors compensated for a loss of mGlu5-regulation of NMDA receptor currents, however: whereas mGlu1 receptors regulate NMDA receptor currents by means of src kinase activation, mGlu5 receptors do so by means of a signal cascade that involves protein kinase C and src kinase (Benquet et al., 2002). It was reported by others that preventing Tamalin interactions with mGlu5 receptors results in the increased internalization of the receptor (Timms et al., 2013) and that Tamalin regulates the trafficking of group I mGlu receptors (Sugi et al., 2007). Thus, a possible molecular mechanism underlying the impairment of LTD could comprise altered surface expression of these receptors. This could explain why both DHPG and CHPG failed to facilitate LTD expression in the presence of pep-mGluR-STL.

Pep-mGlu-STL specifically prevented LTD, but not LTP. Whereas LTP was induced by a brief tetanus, LTD was induced by LFS over a period of several minutes. This different sensitivity of LTP and LTD to pep-mGlu-STL may relate to the perisynaptic localization of group I mGlu receptors (Lujan et al., 1996; Nakamura et al., 2002). Prolonged activation of group I mGlu receptors through glutamate spillover onto perisynaptic receptors may comprise a key mechanism for induction of LTD. LTP on the other hand shows a frequency dependency of mGlu receptor-involvement in LTP (Wilsch et al., 1998). Alternatively, the possibility also exists that Tamalin preferentially regulates LTD. Taken in the context of synaptic information storage, these observations are particularly interesting. Whereas both LTP and LTD are involved in the acquisition of hippocampus-dependent memory (Braunewell and Manahan-Vaughan, 2001; Kemp and Manahan-Vaughan, 2004, 2008; Etkin et al., 2006; Goh and Manahan-Vaughan, 2013a), a division of labor exists in terms of the memory components encoded by these forms of plasticity. LTP appears to be intrinsically involved in the encoding of space *per se* (Kemp and Manahan-Vaughan, 2004, 2008), whereas LTD may encode spatial context (Manahan-Vaughan and Braunewell, 1999; Goh and Manahan-Vaughan, 2013a; Hoang et al., 2018). Group I mGlu receptors are particularly involved in hippocampus-dependent memory encoding (Manahan-Vaughan and Braunewell, 2005; Mukherjee and Manahan-Vaughan, 2013), however, it is not yet clear if specific elements of memory are associated with the specific involvement of particular mGlu receptor subtypes.

## Conclusions

Tamalin subserves glutamate receptor clustering as well as the coupling of mGlu receptors to their signaling proteins (Craven and Bredt, 1998; Sheng and Sala, 1998; Garner et al., 2000). In agreement with the regulation by Tamalin of intracellular trafficking and membrane expression of group I mGlu receptors (Sugi et al., 2007; Timms et al., 2013), as well as its intrinsic role in group I mGlu receptor signaling (Craven and Bredt, 1998; Kitano et al., 2002, 2003), our data support that this PDZ interaction plays a role in group I mGlu receptor-mediated synaptic transmission. These findings add important new insights to previous reports indicating a role for Tamalin in the regulation of group I mGlu receptor function in neurons. The data presented here, comprise the first evidence that Tamalin is intrinsically involved in the signaling cascade required for induction of hippocampal LTD. This finding strongly supports that the PDZ interaction between Tamalin and group I mGlu receptors, and most particularly mGlu5, is required for this form of synaptic plasticity.

## Ethics Statement

The present study was carried out in accordance with the European Communities Council Directive September 22nd, 2010 (2010/63/EU) for care of laboratory animals and after approval of the ethics committee of the local (state) government (Landesamt für Naturschutz, Umweltschutz und Verbraucherschutz, Nordrhein Westfalen).

## Author Contributions

DM-V, K-HB and KD designed the study, and wrote the manuscript that was approved by all authors. SN and DM-V conducted the electrophysiology experiments. DM-V implemented the *in vivo* treatments and performed the electrophysiology analyses. K-HB conducted the neuronal culture experiments. KD generated the peptides. KO’C did the pull-down array experiments. The authors generated the figures respective to their experimental contributions.

## Conflict of Interest Statement

The authors declare that the research was conducted in the absence of any commercial or financial relationships that could be construed as a potential conflict of interest.
